# Surface-Active Catalysts for Interfacial Gas–Liquid–Solid Reactions

**DOI:** 10.1021/accountsmr.5c00026

**Published:** 2025-05-09

**Authors:** Kang Wang, Badri Vishal, Marc Pera-Titus

**Affiliations:** Cardiff Catalysis Institute, 2112 Cardiff University, Cardiff CF10 3AT, United Kingdom

## Abstract

Multiphase reactions combining gas and liquid phases and a solid catalyst are widespread in the chemical industry. The reactions are typically affected by the low gas solubility in liquids and poor mass transfer from the gas phase to the liquid, especially for fast reactions, leading to much lower activity than the intrinsic catalytic activity. In practice, high pressure, temperature, and cosolvents are required to increase the gas solubility and boost the reaction rate. Gas–liquid–solid (G-L-S) microreactors based on particle-stabilized (Pickering) foams rather than conventional surfactant-stabilized foams can increase the contact between the gas and liquid phases, together with surface-active catalytic particles, and dramatically accelerate G-L-S reactions. Unlike surfactants, surface-active catalytic particles can be recycled and reused and reduce coalescence, Ostwald ripening, and aggregation by adsorbing selectively at the G-L interface, promoting stability.

In this Account, we present first a taxonomy of microstructured G-L-(S) interfaces to build G-L-S microreactors (catalytic membrane contactors, microdroplets, micromarbles, microbubbles, and particle-stabilized bubbles/foams). Within this taxonomy, we provide a critical appraisal of surface-active catalytic particles to engineer particle-stabilized aqueous and oil foams. We address the fundamental thermodynamics and dynamics aspects of particle adsorption at the G-L interface and examine the foaming stabilization mechanisms. We further enumerate the possible interactions between particles and G-L interfaces and elucidate how the interfacial self-assembly of surface-active particles can discourage foam destabilization mechanisms. We also discuss strategies for the synthesis of surface-active particles, including surface modification of preformed hydrophilic particles, synthesis of organic–inorganic hybrids, coprecipitation, and bottom-up synthesis, including methods for depositing catalytic centers. Various types of particles capable of stabilizing foams are identified including silica particles modified with hydrophobic and hydrophilic chains, silica particles functionalized with oleophobic and oleophilic chains, biphenyl-bridged organosilica particles, and surface-active polymers. Finally, we highlight recent advances from our group, including catalytic oxidation, hydrogenation, and tandem reactions, facilitated by tailor-designed surface-active particles in aqueous/nonaqueous foam. The relationship between the structure, properties, and foaming performance of surface-active particles, along with their catalytic efficiency within foams, is elucidated. It is our hope that this Account will inspire innovative designs of surface-active particles with tailored properties for the advancement of industrially relevant multiphase reactions. Looking ahead, developing data-driven computational tools would be highly beneficial, allowing the *in silico* design of particles with tailored foaming, foam stability, and local G-L miscibility for defined G-L systems, thus precluding trial-and-error approaches. Parameters such as the three-phase contact angle of particles, the line tension, and the optimal particle size and shape to ensure gas regeneration could be modeled and implemented.

## Graphical abstract

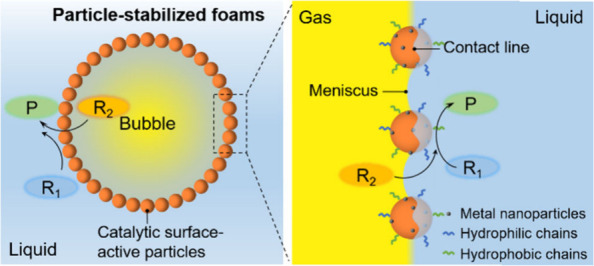

## 1 Introduction

Gas–liquid–solid (G-L-S) reactions, involving gas and liquid reagents and a heterogeneous catalyst, are extensively used in chemical, petrochemical, biochemical, and environmental catalytic processes.^1,2^ State-of-the-art G-L-S reactors comprise packed beds (e.g., trickle beds, bubble columns), stirred tank and bubble column slurry reactors, and fluidized beds.^3–5^ These technologies suffer from low gas solubility in liquids and poor mass and heat transfer of reactants/products to and from the catalyst surface due to the physical separation of the phases. In industrial practice, high gas pressure and temperature, intensive stirring, or the use of surfactants is required to promote the G-L contact and distribute the catalyst between the phases.

Microstructured G-L-(S) interfaces can be engineered to build catalytic G-L-S microreactors that overcome current limitations of state-of-the-art reactors, allowing potential enhancement of reaction rates.^6^ Specifically, G-L-S microreactors enhance mass and heat transfer efficiency and the surface-to-volume ratio by facilitating localized multiphase interactions within a microstructured environment. Recently, we have classified G-L-S microreactors into five families:^7^ (i) catalytic membrane contactors, (ii) microdroplets, (iii) micromarbles, (iv) microbubbles (including cavitation bubbles), and (v) particle-stabilized bubbles (foams). Particle-stabilized bubbles and foams emerge as candidates of choice owing to their versatility and easy implementation to re-engineer state-of-the-art G-L-S reactors. As a key advantage, foams do not require preformed porous membranes as in the case of membrane contactors, reducing the cost. Also, unlike microdroplets and micromarbles, foams can be generated without intricate workup and can be stabilized through straightforward mechanical stirring, which also requires lower energy utilization compared with ultrasonication methods used in microbubble systems.

The engineering of G-L-S microreactors based on bubbles/foams requires surface-active particles with suitable size, distribution, and surface density of hydrophilic–hydrophobic/​oleophilic–oleophobic groups and catalytic centers. Fine control of the particle design can facilitate the location and orientation of catalytic centers at the G-L interface. It can also promote gas regeneration near the catalytic centers along the reaction, thus enhancing the local G-L miscibility and, in turn, tuning the catalytic activity and selectivity.^8^ Despite these benefits, foams have been traditionally regarded as unsuitable for G-L-S reactors involving finely divided catalyst particles due to concerns related to operational instability, inaccurate liquid level control, and the risk of product contamination.^9^ As a matter of fact, dry foams with a polyhedral structure can generate on top of reactors (Figure 1a_1_) that can reduce the accessible volume, lead to poor mixing, and accumulate on the catalyst surface, obstructing the access of reactants to active sites and thereby diminishing mass transfer (see more details in section 3).^10,11^ Industrial operation of G-L-S reactors typically requires the use of defoamers that are typically based on silicones, mineral oils, or hydrophobic solids.^12^ G-L-S microreactors based on particle-stabilized bubbles (bubbly liquids and wet foams) (Figure 1a_2_) can potentially overcome these limitations by locating surface-active catalysts at the G-L interface. This can improve the reactor hydrodynamics and access of reactants to the catalyst surface, provided that the gas in the bubbles can be regenerated so that the reaction is not gas limited.

**Figure 1:**
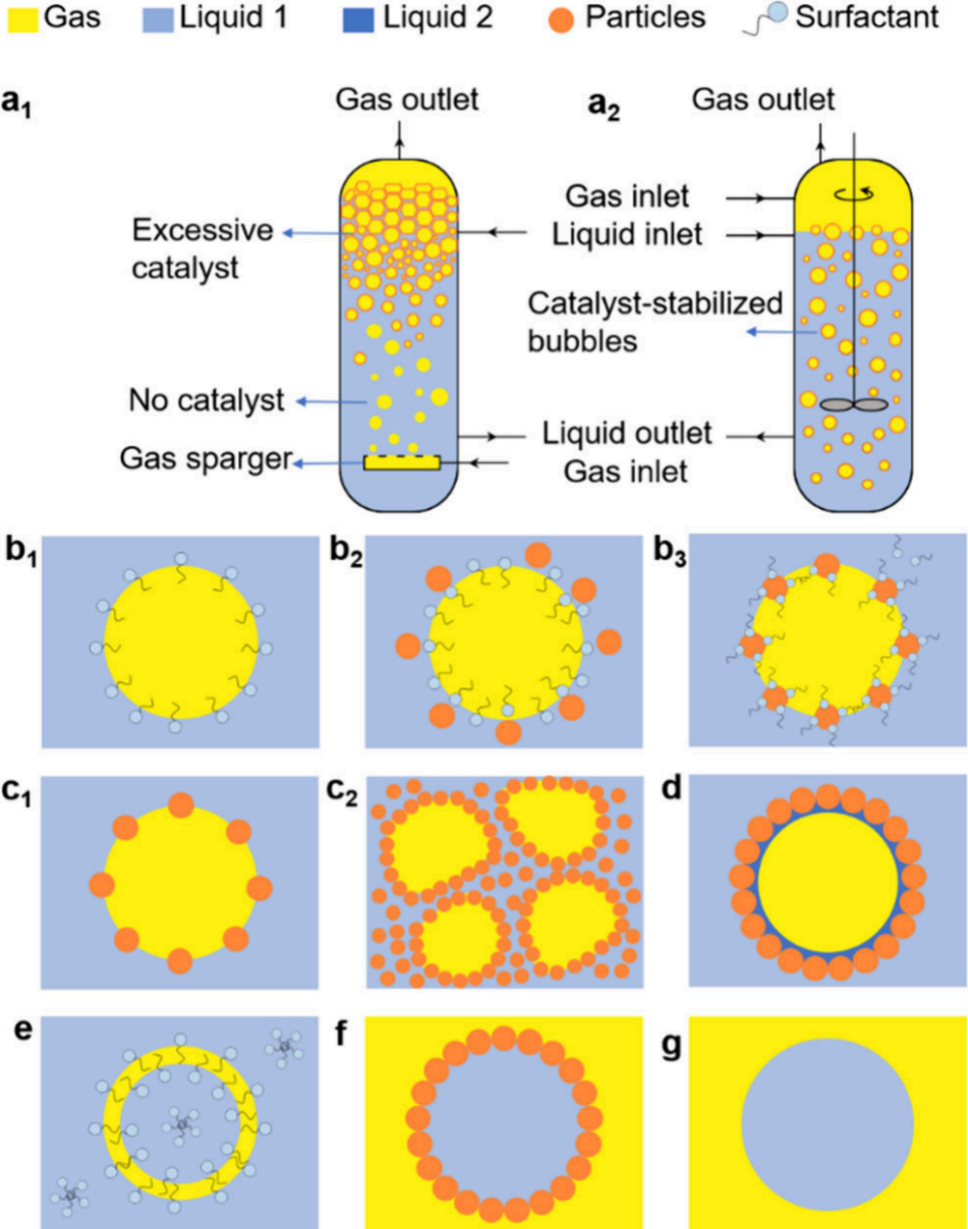
Scheme of (a_1_) state-of-the-art bubble column reactor showing the potential formation of a dry foam on top obstructing the catalytic performance and (a_2_) bubble column reactor implemented with G-L-S microreactors based on particle-stabilized bubbles (bubbly liquid, wet foam). Representation of gas bubbles stabilized by surfactants adsorbed at the G-L interface, either alone (b_1_), surfactants with particles but without interfacial interaction (b_2_), or surfactants combined with particles with interfacial interaction (b_3_). (c) Particles, either self-assembled at the G-L interface (c_1_) or forming a network exceeding the interface (c_2_). (d) Capillary bubbles stabilized by particles with a layer of an insoluble liquid. (e) Antibubbles stabilized by surfactants. (f) Particle-stabilized liquid marble. (g) Microdroplet dispersed in a gas (e.g., spray).

In this Account, we provide a critical appraisal on the design of surface-active catalytic particles to engineer G-L-S microreactors based on aqueous and oil foams and the key drivers to control their self-assembly and location at the G-L interface. We also highlight recent examples reported by our group of applications of surface-active catalytic particles to engineer G-L-S microreactors based on bubbles and foams in water and organic solvents and their credentials for re-engineering already established multiphase reactors to make them more sustainable.

## 2 Particle-Stabilized Bubbles

Bubbles are globular bodies of gas in a liquid. Within this general definition, bubbles can be broadly classified as a function of their size (*D*_G_) as macrobubbles (*D*_G_ > 50 μm), microbubbles (1 < *D*_G_ < 50 μm) and nanobubbles (*D*_G_ < 1 μm). Macrobubbles have strong buoyancy and low stability and have poor applications. In contrast, micro/nanobubbles are encountered in a variety of applications in medicine, industry, water treatment, and food technology. Their most distinguishable features are a reduced buoyancy in solution (i.e., low rising speed), and short-time stability due to the high energy involved to generate the G-L interfacial surface area. Hard H-bonding at the G-L interface can reduce gas diffusion from micro/nanobubbles to the bulk liquid and maintain the kinetic balance against the high internal pressure.^13,14^ Also, the negative charge of micro/nanobubbles under a wide pH range due to interfacial adsorption of HO^–^ anions enhances their stability.^15,16^ Overall, such phenomena hinder gas diffusion from the bubbles to the liquid, allocating an adequate kinetic balance against high internal pressure.

Surfactants can stabilize bubbles, typically in water, by reducing the surface tension and forming a dynamic, flexible interfacial film (Figure 1b_1_). However, surfactants can be hardly recycled, making their use not circular. Particles can also self-assemble at the G-L interface, “armoring” gas bubbles (typically microbubbles) that create a rigid and mechanical barrier preventing their coalescence. Three different shells can be in principle designed: (1) particles embedded into lipid/polymer-stabilized bubbles without adsorbing at the G-L interface by creating a stabilizing network that accumulates at Plateau borders, acting as cork that prevents drainage (Figure 1b_2_) (*vide infra*); (2) combinations of particles and a lipid, polymer, surfactant, or surface-active reagent at the G-L interface (Figure 1b_3_); and (3) single particles or particle aggregates sitting alone at the G-L interface (Figure 1c_1_).^17^ Type (3) shells offer great flexibility to engineer G-L-S reactions and are considered in this Account. In such systems, surface-active particles adsorb at the G-L interface since desorption energies are orders of magnitude higher than thermal fluctuations.^18^ If the particle density at the G-L interface is sufficiently high, particles can generate a rigid “armor” or membrane that is able to not only inhibit gas dissolution/disproportionation but also help adjust the bubble size distribution and prevent neighboring bubbles from coalescence.^19,20^ Additional stabilization can occur by the formation of a particle network between adsorbed and nonadsorbed particles, avoiding liquid drainage (Figure 1c_2_).^21^ Particle armors can maintain anisotropic surface stresses, and therefore, bubbles do not need to be spherical at equilibrium.^22,23^ The particle dynamics can be tuned by the properties of surface-active particles (i.e., nature of intermolecular interactions) that affects their stability at the G-L interface and adsorption kinetics from the bulk liquid (see section 3).

The interfacial stability of particle-stabilized bubbles can be further promoted by incorporating a second fluid phase. Capillary bubbles (or foams) consist typically of particle-stabilized bubbles in water incorporating a minimal amount of oil (as little as 0.1 wt %) that adsorbs at the G-L interface (Figure 1d).^24^ Owing to the higher polarity of oils compared to gases, water-dispersible particles generally exhibit higher affinity for oil–water than for gas–water interfaces, making them effective for stabilizing oil-coated bubbles in water. Related to capillary bubbles, antibubbles are objects characterized by a liquid core encased within a thin air film or shell, surrounded by a bulk liquid medium. Originally termed “inverted” or “inverse” bubbles, antibubbles display two G-L interfaces: one with the inner liquid and another with the outer liquid (Figure 1e).^25,26^ Antibubbles can be stabilized by particles increasing their stability up to several hours.^27^ Particle-stabilized antibubbles can be generated by coating aqueous droplets with hydrophobic colloidal particles, solidifying the droplets, and subsequently introducing them into an aqueous colloidal suspension.

Micromarbles are formed by assembling surface-active (catalytic) particles, which may be hydrophobic or oleophilic and sized in the range 50–1000 nm, at the G-L interface of microdroplets (Figure 1f). This assembly can mitigate liquid evaporation compared to uncoated microdroplets and localize catalytic sites at the G-L interface. As a matter of fact, it is known that microdroplets (1–100 μm) with microstructured G-L interfaces, typically produced using a nebulizer at high gas pressure, functioning as either (electro)­sprays or being deposited on hydrophobic substrates (Figure 1g), can exhibit distinctive features compared to bulk G-L interfaces, leading to a series of nanoscopic phenomena.^7^ For instance, the acidity and basicity can be significantly enhanced at the G-L interface of microdroplets/microbubbles compared to the bulk liquid phase, likely due to restricted hydration, which has important implications for acid–base-catalyzed reactions.^28^ Interfacial assembly of hydrophobic particles can facilitate G-L mixing by creating a gas film between the particles and the liquid. Recent experimental and computational studies have revealed enhanced surface electric fields, on the order of 10^9^ V/m, arising at microscale G-L interfaces due to the preferential adsorption of OH^–^ species.^29^ These fields can make microdroplets/microbubbles function as electrochemical “nanocells”, promoting the generation of HO• radicals and carbocations.

## 3 Particle-Stabilized Foams: Intermolecular Interactions Driving Stabilization

Liquid foams are G-L dispersions, where gas bubbles are dispersed within a liquid medium. Foams typically consist of a collection of gas bubbles separated by thin liquid films, forming a network or matrix. A taxonomy has been established for aqueous foams stabilized by surfactants that can be extrapolated to other liquids and stabilizers.^30^ As a rule, the gas volume fraction or gas holdup (Φ_G_) in the G-L system determines the foam architecture and the interaction between bubbles. At low gas fractions (Φ_G_ < 0.64), commonly found in state-of-the-art G-L-S multiphase reactors (typically Φ_G_ < 0.30 for bubble columns), the G-L system represents a bubbly liquid with spherical bubbles (Figure 2a_1_). At higher gas fractions, a random packing of monodisperse bubbles is generated that exhibits viscoelastic behavior with an apparent yield stress at a critical gas fraction (Φ_G_ ≈ 0.64 for water using surfactants) corresponding to the maximum packing fraction. Foams with 0.64 < Φ_G_ < 0.85 are classified as “wet foams” (Figure 2a_2_). At higher gas fractions, bubbles become increasingly deformed, with curved films between them generating polyhedral shapes. Foams with Φ_G_ > 0.95 are categorized as “dry foams” and are constituted by polyhedral bubbles with a cellular architecture that is described by Plateau’s rules (Figure 2a_3_). As mentioned above, dry foams can occur at the outlet of G-L-S multiphase reactors (e.g., on top of bubble columns) and are often detrimental to their operation (Figure 1a).

**Figure 2:**
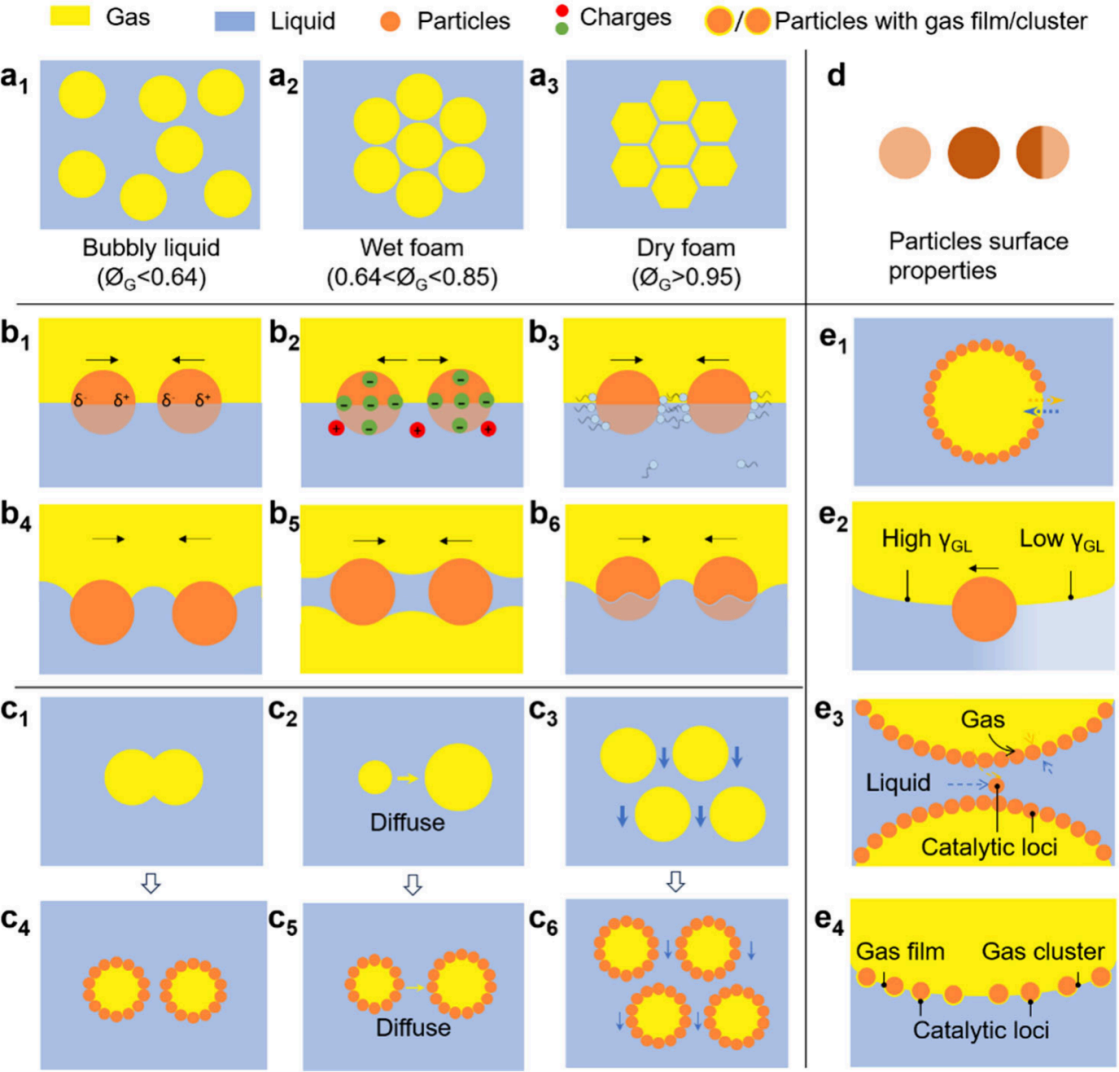
(a_1_–a_3_) Representations of different foam morphologies. Interactions between colloidal particles at G-L interface: (b_1_) van der Waals, (b_2_) electrostatic, (b_3_) hydrophobic, (b_4_) flotation, (b_5_) immersion, and (b_6_) capillary. Foam destabilization mechanisms: (c_1_, c_4_) coalescence, (c_2_, c_5_) coarsening, and (c_3_, c_6_) drainage of liquid. (d) Surface properties of particles. Representation of particles films at the G-L interface: (e_1_) holes or defects in particle film, (e_2_) Marangoni-driven flows, (e_3_) catalytic loci within the thin liquid film between adjacent bubbles and within nearby adsorbed particles, and (e_4_) catalytic loci on self-assembled particles surrounded by gas layers or clusters.

The ability of particles to adsorb at the G-L interface is dictated by particle–interface and particle–particle interactions in the particle film. These interactions determine the particle coverage and surface charge of the bubbles. Four intermolecular interactions, either repulsive or attractive, can be at play, which are van der Waals, electrostatic, hydrophobic, and capillary.^31^ Besides, liquid drainage between the particle and interface and liquid flows can contribute to interfacial particle stabilization.

The first and most straightforward interaction is van der Waals, occurring between all atoms and molecules. As a rule, van der Waals interactions are more important in G-L than in L-L dispersions due to higher Hamaker constants, which are about 3.6 × 10^–20^ N m (air–water–air) compared to 1.0 × 10^–20^ N m (oil–water–oil).^32^ van der Waals interactions are attractive between identical bodies but repulsive when two different bodies are separated by a medium with a dielectric constant between that of both interacting bodies.^33^ This is the case when a hydrophilic silica particle approaches the air–water interface.^34^ van der Waals interactions can also occur between particles adsorbed at the G-L interface through either the air or liquid phases (Figure 2b_1_). Attractive van der Waals interactions are stronger in air than in the liquid phase, promoting particle stabilization.^35^ Particle hydrophobization can promote the interfacial interaction between particles and the gas phase, especially in water, resulting in more cohesive particle films.^36^

Electrostatic interactions occur when the electrical double layers of the approaching surfaces overlap. They also occur between a charged particle and the G-L interface, especially in the presence of polar solvents with high dielectric constants like water, as the gas–water interface is charged by the adsorption of HO^–^ ions from water, and the particle can come closer to the gas–water interface. The magnitude of electrostatic repulsions increases with the particle and gas–water interface charge and decreases with the ionic strength (Figure 2b_2_).^37^ The location of particles or surfactants at the G-L interface can alter the interaction pattern between a particle in suspension and the interface that results in changes in the interfacial properties (e.g., charge, deformability). For instance, long-range attraction can occur between a negatively charged particle in the aqueous phase and an air–water interface with adsorbed cationic surfactants.^38^ The approach of a negatively charged particle to a monolayer of cationic surfactants at the interface can even result in the monolayer being transferred to the particle due to attractive electrostatic interactions.^39^ Electrostatic interactions can also occur between charged particles adsorbed at the air–water interface, where the particle charge resides only on the particle surface that is in contact with the aqueous phase.^40^ Long-range electrostatic interactions can be at play between two charged particles at the air–water interface, whereas electrostatic repulsions can stabilize colloidal suspensions against aggregation.^41^ The structuring of particle monolayers at the air–water interface is very sensitive to the electrolyte concentration. At low electrolyte concentration, ordered structures resulting from interparticle repulsion can be observed using charged polystyrene particles, while at high electrolyte concentration, the particles form 2D clusters.^35^

Electrostatic interactions between particles can be screened by electrolytes in the aqueous phase at high ionic strength, leading to spontaneous particle agglomeration. This occurs because the high ionic strength compresses the electrical double layer surrounding each particle, reducing repulsive forces that normally keep them apart. As a result, attractive van der Waals interactions dominate, causing the particles to assemble and aggregate spontaneously.

Hydrophobic interactions between a particle and an interface can also occur due to the formation of capillary bridges, which are magnified for polar liquids with high surface tensions (e.g., water).^42^ Stable liquid films can be formed between a particle and the interface if repulsive van der Waals interactions are stronger than hydrophobic interactions (Figure 2b_3_).^43^ Attractive hydrophobic interactions can be promoted in three circumstances: (1) by suppressing particle–interface electrostatic repulsions at lower interfacial charges, (2) by increasing the ionic strength in polar solvents or using apolar solvents, and (3) by implementing hydrophobized particles.^43–45^ The presence of surfactants adsorbed at the G-L interface can promote hydrophobic interactions, especially for surfactants with longer alkyl chain lengths. The interaction between particles and surfactants can be driven by electrostatic attraction when charged surfactants interact with charged particles. Surfactants with longer hydrocarbon chains tend to exhibit stronger hydrophobic interactions. Surfactants can adsorb on particle surfaces, which alters their hydrophobicity. The hydrophobic interaction with a suitable degree between the surfactants and particles prevents bubble coalescence and Ostwald ripening, thus maintaining the foam structure and stability. Interparticle attractions can also be increased by introducing attractive hydrophobic interactions between the particles.^46^

Capillary interactions can occur when a particle meets a G-L interface by forming a meniscus and a contact line around a particle (Figure 2b_4_–b_6_). These interactions are affected by the surface tension due to the interface curvature and are affected by the nature and composition of the liquid and the particle properties such as the charge, hydrophobicity, size, shape (e.g., spheres, nanofibers, nanotubes, nanosheets), porosity (e.g., porous superparticles), and roughness that condition the interfacial packing of particles.^47^ When a three-phase contact line is formed between a particle and an interface, capillary interactions pull the particle into the gas. Capillary interactions can also occur between particles adsorbed at the G-L interface, promoting film stabilization and stiffness resulting in interfacial deformation.^40^ Capillary interactions can be promoted at lower interparticle spacing with a concomitant surface pressure increase. A consequence of capillary interactions is the formation of a thin gas layer on the adsorbed particles. This layer can increase the G-L miscibility and thus generate *loci* that can enhance the rate of reactions at the interface. The dynamic, continuous renewal of this gas layer during a reaction is a necessary condition to ensure that the gas is not a limiting reactant in the vicinity of catalytic centers during a reaction.

Foams can destabilize and segregate with time due to three main phenomena: (1) coalescence (film rupture) (Figure 2c_1_,c_4_), (2) “coarsening” (gas diffusion between bubbles with different Laplace pressures) (Figure 2c_2_,c_5_), and/or (3) “drainage” of liquid (Figure 2c_3_,c_6_). These phenomena are analogous to those observed in emulsions, with the primary distinction being that coarsening is referred to in emulsions as Ostwald ripening. Coalescence involves the rupture of films between bubbles that is influenced by factors such as hydrodynamics, surface rheology, surface forces, and thermal fluctuations. Particles can stabilize foams by forming a protective liquid layer between adjacent bubbles, promoting enhanced steric and electrostatic repulsions.^48,49^ Coarsening comprises the growth and shrinkage of bubbles driven by gas diffusion between adjacent bubbles. The driving force for coarsening is the Laplace pressure or pressure difference between the interior and exterior of bubbles. The rate of coarsening depends on Φ_G_, the average bubble size, and the gas and liquid properties. Finally, drainage refers to irreversible liquid transfer through liquid films driven by gravity and capillary forces. As gravity prompts liquid drainage, the upper part of a foam rapidly dries, increasing the Φ_G_, while the lower section remains moist. Drainage alters the bubble shape, transforming it from spherical to polyhedral. Drainage can be inhibited at higher liquid viscosities and by using particles with smooth surfaces. Upon drainage completion, thin films between bubbles become exceedingly thin (5–20 nm), increasing the likelihood of rupture and bubble coalescence.

Foams generally exhibit a short lifespan without stabilizers, which limits their applications in many contexts. Particles assembled at the G-L interface significantly reduce the rates of coalescence, coarsening, and drainage. Surface-active particles with high recyclability serve as effective foam stabilizers. Particle-stabilized foams with appropriate foamability and stability can form, break, and regenerate under stirring that facilitates the continuous renewal of gas reactants. As a result, G-L catalytic reactions can be continuously sustained within a foam reactor. Overall, these destabilization phenomena, while detrimental to foam stability, can be relevant for catalytic reactions since they can increase the G-L interfacial surface, G-L miscibility near catalytic sites, and gas renewal.

## 4 Surface-Active Particles for Foam Stabilization

### 4.1 Key Drivers to Designing Surface-Active Particles

G-L-S microreactors based on particle-stabilized foams require the engineering of bubbles in aqueous/nonaqueous liquids with microstructured G-L-S interfaces. To this aim, it is crucial to master the particle self-assembly at the G-L interface and the particle dynamics under reaction conditions. The thermodynamics and dynamics rules driving interfacial particle self-assembly and foam formation are compiled in the Supporting Information.

To design a successful surface-active particle, this requires a fine balance of different interactions that depends on the nature of surface groups (e.g., OH groups, alkyl chains, aromatic groups), their distribution (e.g., random or asymmetric), the liquid and gas properties, and the interfacial architecture (e.g., presence of surface charge and presence of adsorbed molecules) (Figure 2d). These interactions can be altered under a chemical reaction promoting concentration and temperature gradients that can affect the interfacial self-assembly and packing of particles and thus the stiffness of particle films. The presence of holes or defects in the films, which affects the surface coverage of particles, φ_a_, can enhance their lateral motion on the interface (Figure 2e_1_).^50^ As a result, Marangoni-driven flows can be induced by local differences in surface tension at the G-L interface due to concentration gradients of surfactants that can affect the interfacial positioning of particles and thus the architecture of particle films (Figure 2e_2_).^51^ All these elements are instrumental to engineer *loci* with increased G-L miscibility and access to catalytic sites to enhance catalytic reactions. These *loci* can be located within the thin liquid film between adjacent bubbles (Figure 2e_3_), within nearby adsorbed particles at the G-L interface or as a gas layer or gas clusters within the interparticle space or on the rough surfaces of particles (Figure 2e_4_). The architecture of the particle layer is also instrumental to promoting gas regeneration along the reaction. As a matter of fact, since the gas density is about 3 orders of magnitude lower than that of liquids, the reaction can be easily inhibited in the presence of very compact particle films.

### 4.2 Synthesis of Surface-Active Particles

To tailor-design surface-active particles, it is necessary to adjust the surface composition and distribution of surface functions (e.g., random or asymmetric) on the particle surface. Also, a particle size in the range 200–500 nm is necessary to ensure sufficient adsorption strength for particle self-assembly combined with fast particle diffusion from the bulk liquid to the G-L interface.

Surface-active catalytic particles can be prepared using a variety of methods including postgrafting, coprecipitation, and bottom-up synthesis. The postgrafting method involves the surface modification of preformed particles using typically organosilanes with varying degrees of hydrophobicity or organic acids of different chain lengths.^52^ Surface modification occurs by the formation of chemical bonds through hydrolytic condensation, providing high particle stability. Metal oxide particles are commonly used owing to the presence of hydroxyl groups that facilitate surface modification. Examples include, among others, SiO_2_, TiO_2_, CeO_2_, and Fe_3_O_4_. Pure metal particles can sometimes generate self-assembled monolayers with thiol-containing organic compounds, thereby exhibiting surface activity.^53^ Surface-active particles can be further functionalized with metal nanoparticles as catalytic centers to enhance the catalytic activity. Typically, metal nanoparticles are loaded using methods such as impregnation, the sol–gel method, or deposition–reduction. The coprecipitation method is used to synthesize surface-active particles where the organic precursors in solution are simultaneously precipitated by adding a precipitating agent, often under controlled pH and temperature.^54^ This method enables uniform mixing of components at the molecular level, making it widely used for the synthesis of surface-active particles. Core–shell architectures can be generated by the sequential addition of different precursors. The bottom-up synthesis method has been employed to prepare Janus-structured particles from precursors, such as tetraethyl orthosilicate. Through condensation of the precursors, particles are formed, which can be subsequently modified to achieve the desired surface anisotropy and wetting properties.^8^ The advantages and disadvantages of different methods for synthesizing surface-active particles are listed in Table S1.

## 5 Examples of Catalytic G-L-S Microreactors Based on Foams

A few groups have recently investigated particle-stabilized foams as G-L-S microreactors for catalysis. Huang et al. applied this concept for the selective oxidation of alcohols with air. The authors prepared hybrid organic–inorganic particles by self-assembly between a rigid tripodal ligand and polyoxometalate anions, and Au nanoparticles were further embedded on the particles. A given amount of an alcohol was added into the aqueous dispersion of particles that could generate foams in the presence of microbubbles that promoted interfacial alcohol oxidation. Huang et al. designed pH-responsive aqueous foams stabilized by partially hydrophobized silica particles containing hydrophilic triamine and hydrophobic octyl groups and Pd and Au nanoparticles.^52^ The hydrophobicity was finely tuned by varying the molar ratio of the triamine and octyl groups. The foamability exhibited a maximum with a triamine-to-octyl molar ratio of 0.66. The foam significantly enhanced the catalytic activity in hydrogenation and oxidation reactions compared to the reactions in bulk water. Foams were destabilized after the reaction by changing the pH, allowing catalyst separation from the reaction product and reuse (Figure 3a_1_,a_2_).

**Figure 3:**
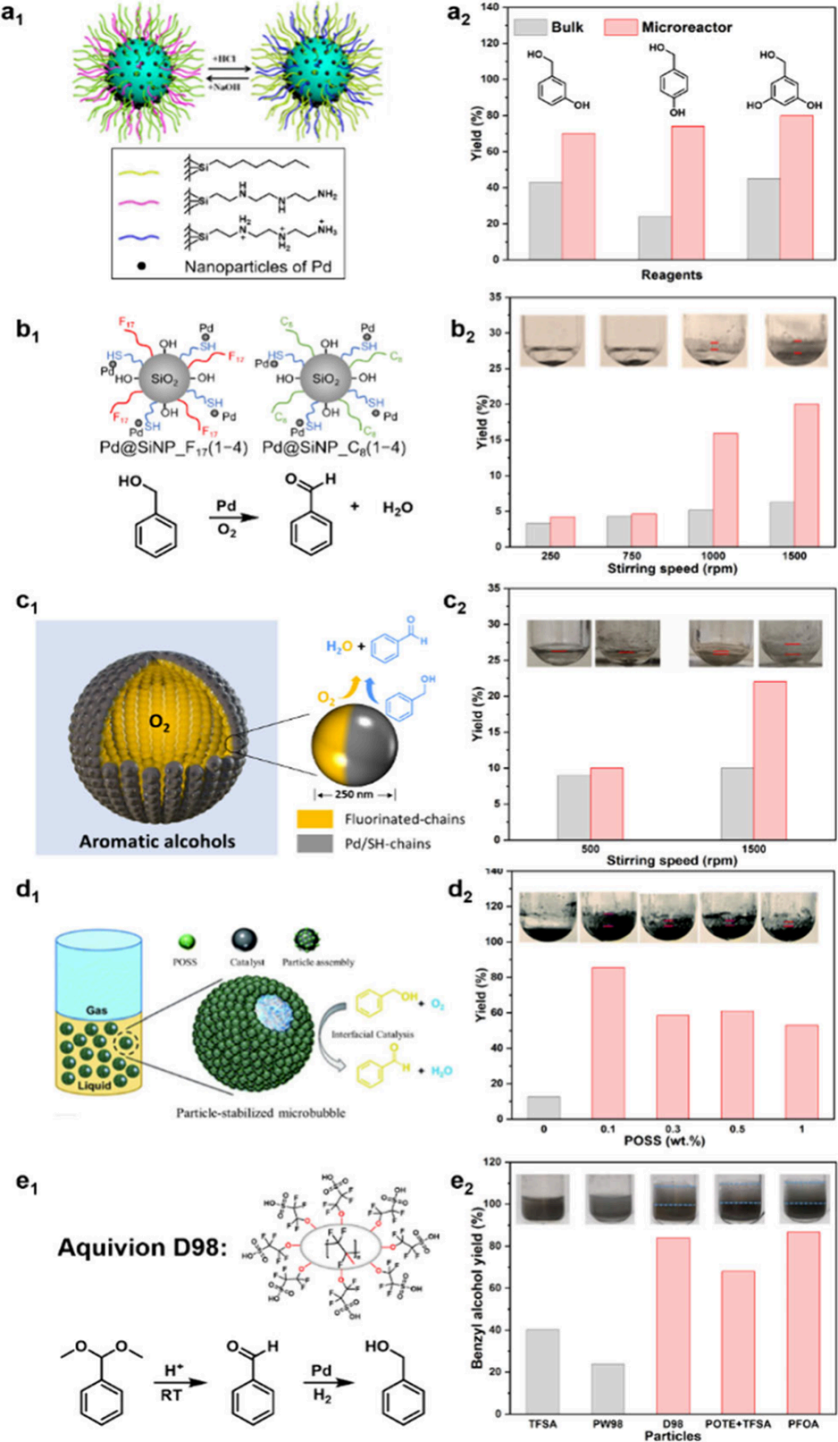
Aqueous foams for catalysis: (a_1_) structure of pH-responsive, hydrophobic particles allowing protonation/deprotonation and (a_2_) oxidation of aromatic alcohols in bulk and foam. 5 mL of water, 0.5 mmol of substrate, 5 wt % catalyst, 900 rpm, 50 or 80 °C, 5 or 8 bar O_2_, 5–9 h. Images reproduced with permission from ref 52. Copyright 2015 American Chemical Society. Oil foams for catalysis: (b_1_) structure of Pd@SiNP_F_17_(1–4) and Pd@SiNP_C_8_(1–4) particles, (b_2_) aerobic oxidation of BnOH in bulk and foam, and foamability in 1.8 mL of BnOH/xylene (1:1 v/v) against the stirring rate at 80 °C, 1 h, 1 wt % particles, 1 bar air. Images reproduced with permission from ref 54. Copyright 2022 American Chemical Society. Oil foams stabilized by Janus particles for catalysis: (c_1_) representation of Janus particle and oil foam and (c_2_) aerobic oxidation of BnOH in bulk and foam at variable stirring rate. 1.8 mL of BnOH/xylene (1:1 v/v), at 100 °C, 1 h, 1 wt % particles, 1 bar O_2_. Images reproduced with permission from ref 8. Copyright 2024 American Chemical Society. Oil foams stabilized by dual-particle system for catalysis: (d_1_) representation of POSS/silica dual-particle system and (d_2_) aerobic oxidation of BnOH over Pd@SiNP_F_17_ at variable Ph_7_/F_13_-POSS concentration at 80 °C, 2 h, 2 wt % Pd@SiNP_F_17_, 0.1 wt % Ph_7_/F_13_-POSS, 1500 rpm. Images reproduced with permission from ref 55. Copyright 2022 Royal Society of Chemistry. Oil foams stabilized by dual-particle system for one-pot tandem catalysis: (e_1_) structure of Aquivion D98-20BS-P, (e_2_) BnOH yield in tandem deacetalization-hydrogenation of benzaldehyde dimethyl acetal, and foamability at 10 wt % solid acid, 10 mg of Pd/SiO_2_, 0.5 mmol of reactant, 2 mL of H_2_O, 1.5 bar H_2_, room temperature, 700 rpm. Images reproduced with permission from ref 56. Copyright 2022 Wiley.

Recently, we have demonstrated that particles containing phenyl rings and alkyl chains can assemble at the air–liquid interface, stabilizing foams based on aromatic solvents while eliminating the need for fluorinated chains.^57^ Besides, we have designed oil foams stabilized by surface-active catalytic particles bearing fluorinated chains and Pd nanoparticles [Pd@SiNP_F_17_(1–4)], allowing fast and efficient aerobic oxidation of a variety of aromatic and aliphatic alcohols at ambient O_2_ pressure compared to bulk catalytic systems.^54^ The fluorinated chains tuned the wettability, while Pd nanoparticles acted as catalytic centers. For comparison, a Pd@SiNP_C_8_(1–4) catalyst without surface-active properties was prepared that dispersed in the bulk liquid (Figure 3b_1_). The catalytic performance was affected by the foaming properties, with an 8 times activity increase with Pd@SiNP_F_17_(1–4) (with foams) compared to Pd@SiNP_C_8_(1–4) (without foam) for benzyl alcohol (BnOH) oxidation.^54^ Without foam, both particles exhibited a comparable catalytic performance (Figure 3b_2_). At a stirring rate in the range of 750–1000 rpm, Pd@SiNP_F_17_(1–4) afforded a significant increase in the benzaldehyde (BAH) yield after 1 h of reaction, whereas the yield for Pd@SiNP_C_8_(1–4) remained almost unchanged. At higher stirring rates (1000–1500 rpm), the BAH yield for Pd@SiNP_F_17_(1–4) increased steadily, which was associated with an expanded foam volume.

Silica Janus particles were designed to conduct aerobic oxidation reactions in nonaqueous foam.^8^ A Stöber silica core was grafted selectively with fluorinated and mercaptopropyl chains on each hemisphere, enabling tunable adjustment of oleophobic–oleophilic properties. The particles were decorated with Pd nanoparticles in the oleophilic hemisphere (Pd/JPs). A non-Janus catalyst (Pd/non-JPs) was prepared for comparison. The catalysts were implemented in the aerobic oxidation of BnOH in a BnOH/*o*-xylene (1:1 v/v) mixture at 100 °C for 1 h with stirring rates ranging from 500 to 1500 rpm (Figure 3c_1_,c_2_). Under nonfoaming conditions (500 rpm), both catalysts exhibited a similar BAH yield (∼9%). However, at 1500 rpm, Pd/JPs exhibited much higher yield (22%), while Pd/non-JPs showed little change (∼9%). This marked difference was attributed to foam generation by Pd/JPs at 1500 rpm, whereas Pd/non-JPs showed poor foamability.

By combining novel Ph_7_/F_13_-POSS particles, used as a frother, and surface-active catalytic organosilica particles (Pd@SiNP_F_17_), used as a stabilizer, a dual-particle system was designed that generated foams in pure BnOH for aerobic oxidation (Figure 3d_1_).^55^ Without Ph_7_/F_13_-POSS, the BAH yield was only 12% at 80 °C for 2 h and 2 wt % Pd@SiNP_F_17_, but it increased to 85% by adding 0.1 wt % Ph_7_/F_13_-POSS (Figure 3d_2_). This improvement was attributed to a higher dispersion of Pd@SiNP_F_17_ particles, along with the foam formation and transfer of catalytic particles from bulk BnOH to the G-L interface. The BAH yield declined at Ph_7_/F_13_-POSS in the range 0.3–1.0 wt % due to foam instability (Figure 3d_2_). At higher concentration, Ph_7_/F_13_-POSS predominantly occupied the interface, shifting the catalytic Pd@SiNP_F_17_ particles into bulk BnOH. These “armored” G-L interfaces exhibited a reduced permeability that decreased O_2_ renewal along the reaction.

Aquivion perfluorosulfonic acid (PFSA) is a superacid resin with surface-active properties for stabilizing oil-in-water emulsions. Aquivion D98-20BS-P (dispersion) combined with Pd/SiO_2_ was employed to design foams for one-pot tandem deacetalization-hydrogenation reactions using benzaldehyde dimethyl acetal as a reactant (Figure 3e_1_).^56^ H-bond interactions between Aquivion D98-20BS-P and solvent molecules (e.g., BnOH, aniline, and water) promoted the foamability. By combining Aquivion D98-20BS-P and a Pd/SiO_2_ catalyst, the tandem reaction reached an overall BnOH yield of 82% with no toluene formation (Figure 3e_2_). Control experiments combining trifluoromethanesulfonic acid (TFSA) and Pd/SiO_2_, without foam formation, afforded a BnOH yield of only 40% after 20 min. Likewise, using Aquivion PW98 (solid acid powder) afforded only a 20% yield with no foam formation. Incorporating the surfactant polyoxyethylene (10) tridecyl ether (POTE) into the system enabled foam generation, increasing the yield to 69%. Also, combining perfluorooctanoic acid (PFOA) with Pd/SiO_2_ resulted in a higher BnOH yield (87%) but led to the formation of toluene as a byproduct with 10% yield.

In the aforementioned catalytic reactions, foams provide a microstructured catalytic environment in which surface-active particles at G-L interfaces form distributed, accessible, and reactive sites, while the foam architecture structure itself enhances mass transfer, local gas concentration, and catalyst density at the interface. The unique properties of the surface-active particles contribute to the catalytic performance in ways that conventional systems often cannot replicate.

## 6 Conclusions and Future Directions

In this Account, we have summarized recent developments of G-L-S microreactors stabilized by surface-active catalytic particles as a platform to adjust the microenvironment of catalytic reactions. Surface-active catalytic particles enable the development of sustainable, efficient, and scalable G-L-S microreactors for a wide range of chemical reactions. The studies presented in this Account highlight how particle-stabilized foams and bubbles address traditional challenges of G-L-S reactors, such as limited gas solubility and inefficient mass transfer, by utilizing tailored surface-active particles with adjustable wettability, surface chemistry, and morphology to stabilize G-L interfaces and enhance the catalytic performance. Unlike conventional (hydrophilic) catalysts, surface-active catalysts, particularly those functioning in Pickering foams or interfacially assembled systems, provide a paradigm shift in catalyst design from passive bulk dispersion to active localization at microstructured interfaces. These unique properties enable higher efficiency, selectivity, and process intensification in G-L-S catalytic systems, affording milder operation conditions.

The examples reported so far focus on applications of thermal catalysis. Surface-active particles can be expanded to photo-, bio-, and electrocatalysis. For example, commodities such as H_2_O_2_ could be photocatalytically synthesized at the interface of particle-stabilized O_2_ foams using surface-active semiconductor particles. Particles might also be functionalized with enzymes being localized at the G-L interface. Enzymes can exhibit synergistic activity with other catalytic centers in the particles and thus assist in the design of tandem reactions. Surface-active particles could also be used as gas transporters for applications in electrochemistry, while conductive particles can serve as extended electrodes, effectively increasing the electrode surface area and enhancing the reactivity.

A missing gap to date is how to establish relationships between the G-L-S interface microstructure and the nature and strength of particle–particle and particle–interface interactions. This understanding will enable the *in silico* data-driven design of particles, reducing the reliance on time-consuming trial-and-error approaches. To this aim, simulation methods such as dissipative particle dynamics and molecular dynamics can be employed for particle design. These methods, already established for particle-stabilized emulsions, can help elucidate the particle location at the G-L interface and charge, as well as the local G-L miscibility near catalytic centers, molecular orientation, and other underlying interfacial nanoscopic phenomena. The design of environmentally friendly, nonfluorinated catalysts for G-L-S reactions will play a pivotal role in addressing this goal. Also, these methods can assist the design of new, unprecedented G-L-S microreactors based on different microstructured G-L-S interfaces.

G-L-S catalytic microreactors based on particle-stabilized foams show main advantages ascribed to the possibility of re-engineering state-of-the-art G-L-S reactors without major technological changes and the possibility of operating with high gas holdups (>30%). However, G-L-S catalytic microreactors are still at the early stage of development, with reagent volumes being limited by the foaming method. To address this, it is crucial to develop well-adapted reactor hydrodynamics methods for scaling up particle-stabilized foams under reaction conditions and re-engineering state-of-the-art multiphase reactors. In addition, upscaling of surface-active particle synthesis methods, recycling of surface-active particles after operation, and control of adsorption dynamics at the gas–liquid interface to enable gas regeneration during operation, avoiding this to become a limiting reactant, require systematic consideration for industrial development.

## Supplementary Material



## Notes

Thermodynamics and dynamics of particle self-assembly at the G-L interface and foam formation; evolution of adsorption energy of a single particle and free energy of foam formation against the contact angle; and advantages and drawbacks of synthetic methods of surface-active particles (PDF)

## Abbreviations

BnOH: benzyl alcohol
BAH: benzaldehyde
G-L-S: gas–liquid–solid
L-L: liquid–liquid
PFSA: perfluorosulfonic acid
POSS: polyoctahedral silsesquioxane
SiNP: silica nanoparticle

## References

[1] Shah, Y. T. ; Sharma, M. M. , Gas-liquid-solid reactors. In Chemical Reaction and Reactor Engineering; CRC Press, 2020; pp 667–734.

[2] Tan J., Ji Y.-N., Deng W.-S., Su Y.-F. (2021). Process intensification
in gas/liquid/solid reaction in trickle bed reactors: A review. Petroleum Science.

[3] Shah, Y. T. In Gas-liquid-solid reactor design; 1979.

[4] Ramachandran, P. ; Chaudhari, R. In Three-phase catalytic reactors; 1983.

[5] Henkel, K.-D. Reactor types and their industrial applications. In Ullmann’s Encyclopedia of Industrial Chemistry; 2000.

[6] Dedovets D., Li Q., Leclercq L., Nardello-Rataj V., Leng J., Zhao S., Pera-Titus M. (2022). Multiphase
microreactors based on liquid–liquid
and gas–liquid dispersions stabilized by colloidal catalytic
particles. Angew. Chem., Int. Ed..

[7] Wang K., Pera-Titus M. (2024). Microstructured
gas-liquid-(solid) interfaces: A platform
for sustainable synthesis of commodity chemicals. Science Advances.

[8] Wang K., Davies-Jones J., Graf A., Carravetta M., Davies P. R., Pera-Titus M. (2024). Amphiphilic
Janus particles for aerobic
alcohol oxidation in oil foams. ACS catalysis.

[9] Tiso T., Demling P., Karmainski T., Oraby A., Eiken J., Liu L., Bongartz P., Wessling M., Desmond P., Schmitz S. (2024). Foam control in biotechnological processes - challenges and opportunities. Discover Chemical Engineering.

[10] Xu W., Lu Z., Sun X., Jiang L., Duan X. (2018). Superwetting
electrodes
for gas-involving electrocatalysis. Acc. Chem.
Res..

[11] Vijayaraghavan K., Nikolov A., Wasan D. (2006). Foam formation and
mitigation in
a three-phase gas–liquid–particulate system. Advances in colloid and interface science.

[12] Denkov N. D. (2004). Mechanisms
of foam destruction by oil-based antifoams. Langmuir.

[13] Parkinson L., Sedev R., Fornasiero D., Ralston J. (2008). The terminal rise velocity
of 10–100 μm diameter bubbles in water. J. Colloid Interface Sci..

[14] Ohgaki K., Khanh N. Q., Joden Y., Tsuji A., Nakagawa T. (2010). Physicochemical
approach to nanobubble solutions. Chem. Eng.
Sci..

[15] Tian C., Ji N., Waychunas G. A., Shen Y. R. (2008). Interfacial structures of acidic
and basic aqueous solutions. J. Am. Chem. Soc..

[16] Buch V., Milet A., Vácha R., Jungwirth P., Devlin J. P. (2007). Water surface is acidic. Proc.
Natl. Acad. Sci. U. S. A..

[17] Costa C., Medronho B., Filipe A., Mira I., Lindman B., Edlund H., Norgren M. (2019). Emulsion formation
and stabilization
by biomolecules: The leading role of cellulose. Polymers.

[18] Correia E. L., Brown N., Razavi S. (2021). Janus particles at fluid interfaces:
Stability and interfacial rheology. Nanomaterials.

[19] Bala
Subramaniam A., Abkarian M., Mahadevan L., Stone H. A. (2005). Non-spherical bubbles. Nature.

[20] Abkarian M., Subramaniam A. B., Kim S.-H., Larsen R. J., Yang S.-M., Stone H. A. (2007). Dissolution
arrest and stability of particle-covered
bubbles. Physical review letters.

[21] Dickinson E., Ettelaie R., Kostakis T., Murray B. S. (2004). Factors controlling
the formation and stability of air bubbles stabilized by partially
hydrophobic silica nanoparticles. Langmuir.

[22] Kim S.-H., Heo C.-J., Lee S. Y., Yi G.-R., Yang S.-M. (2007). Polymeric
particles with structural complexity from stable immobilized emulsions. Chem. Mater..

[23] Majumder M., Chopra N., Andrews R., Hinds B. J. (2005). Enhanced flow in
carbon nanotubes. Nature.

[24] Zhang Y., Wu J., Wang H., Meredith J. C., Behrens S. H. (2014). Stabilization of
liquid foams through the synergistic action of particles and an immiscible
liquid. Angew. Chem..

[25] Postema M., Novell A., Sennoga C., Poortinga A. T., Bouakaz A. (2018). Harmonic response from microscopic
antibubbles. Applied Acoustics.

[26] Kotopoulis S., Johansen K., Gilja O. H., Poortinga A. T., Postema M. (2015). Acoustically active antibubbles. Acta Phys. Polym., A.

[27] Poortinga A. T. (2011). Long-lived
antibubbles: stable antibubbles through Pickering stabilization. Langmuir.

[28] Mishra H., Enami S., Nielsen R. J., Stewart L. A., Hoffmann M. R., Goddard W. A., Colussi A. J. (2012). Brønsted basicity
of the air–water interface. Proc. Natl.
Acad. Sci. U. S. A..

[29] Xiong H., Lee J. K., Zare R. N., Min W. (2020). Strong electric field
observed at the interface of aqueous microdroplets. journal of physical chemistry letters.

[30] Amani P., Miller R., Javadi A., Firouzi M. (2022). Pickering foams and
parameters influencing their characteristics. Adv. Colloid Interface Sci..

[31] McNamee C. E., Yamamoto S. (2020). Forces between a hard surface and
an air–aqueous
interface with and without films. Curr. Opin.
Colloid Interface Sci..

[32] Coons J., Halley P., McGlashan S., Tran-Cong T. (2005). Scaling laws
for the critical rupture thickness of common thin films. Colloids Surf., A.

[33] Israelachvili, J. N. In Intermolecular and surface forces; Academic Press, 2011.

[34] Tabor R. F., Manica R., Chan D. Y., Grieser F., Dagastine R. R. (2011). Repulsive
van der Waals forces in soft matter: why bubbles do not stick to walls. Physical review letters.

[35] Aveyard R., Clint J. H., Nees D., Paunov V. N. (2000). Compression and
structure of monolayers of charged latex particles at air/water and
octane/water interfaces. Langmuir.

[36] Tolnai G., Agod A., Kabai-Faix M., Kovács A., Ramsden J., Hórvölgyi Z. (2003). Evidence for
secondary
minimum flocculation of stöber silica nanoparticles at the
air– water interface: film balance investigations and computer
simulations. J. Phys. Chem. B.

[37] Englert A., Ren S., Masliyah J., Xu Z. (2012). Interaction forces between a deformable
air bubble and a spherical particle of tuneable hydrophobicity and
surface charge in aqueous solutions. J. Colloid
Interface Sci..

[38] Ren S., Masliyah J., Xu Z. (2014). Studying bitumen–bubble interactions
using atomic force microscopy. Colloids Surf.,
A.

[39] McNamee C. E., Kappl M., Butt H.-J., Ally J., Shigenobu H., Iwafuji Y., Higashitani K., Graf K. (2011). Forces between a monolayer
at an air/water interface and a particle in solution: influence of
the sign of the surface charges and the subphase salt concentration. Soft Matter.

[40] Bleibel J., Domínguez A., Oettel M. (2013). Colloidal particles at fluid interfaces:
Effective interactions, dynamics and a gravitation–like instability. European Physical Journal Special Topics.

[41] McGorty R., Fung J., Kaz D., Manoharan V. N. (2010). Colloidal
self-assembly at an interface. Mater. Today.

[42] Fritzsche J., Peuker U. A. (2016). Modeling adhesive forces caused by nanobubble capillary
bridging. Colloids Surf., A.

[43] Fielden M. L., Hayes R. A., Ralston J. (1996). Surface and
capillary forces affecting
air bubble– particle interactions in aqueous electrolyte. Langmuir.

[44] Cui X., Shi C., Zhang S., Xie L., Liu J., Jiang D., Zeng H. (2017). Probing the effect
of salinity and pH on surface interactions between
air bubbles and hydrophobic solids: implications for colloidal assembly
at air/water interfaces. Chemistry - An Asian
Journal.

[45] Albijanic B., Ozdemir O., Hampton M., Nguyen P., Nguyen A., Bradshaw D. (2014). Fundamental aspects of bubble–particle attachment
mechanism in flotation separation. Minerals
Engineering.

[46] McNamee C. E., Fujii S., Yusa S.-i., Azakami Y., Butt H.-J., Kappl M. (2015). The forces and physical properties of polymer particulate monolayers
at air/aqueous interfaces. Colloids Surf., A.

[47] Chatterjee N., Flury M. (2013). Effect of particle
shape on capillary forces acting on particles
at the air–water interface. Langmuir.

[48] Kralchevsky P. A., Denkov N. D. (2001). Capillary
forces and structuring in layers of colloid
particles. Current opinion in colloid &
interface science.

[49] Denkov N., Ivanov I., Kralchevsky P., Wasan D. (1992). A possible mechanism
of stabilization of emulsions by solid particles. J. Colloid Interface Sci..

[50] McNamee C. E., Kappl M. (2016). Forces and physical
properties of the Langmuir monolayers of TiO_2_ particles
at air/water interfaces after collisions by a particle
in water. RSC Adv..

[51] Sáenz P., Valluri P., Sefiane K., Karapetsas G., Matar O. (2014). On phase change in Marangoni-driven
flows and its effects on the
hydrothermal-wave instabilities. Phys. Fluids.

[52] Huang J., Cheng F., Binks B. P., Yang H. (2015). pH-responsive gas–water–solid
interface for multiphase catalysis. J. Am. Chem.
Soc..

[53] Love J. C., Estroff L. A., Kriebel J. K., Nuzzo R. G., Whitesides G. M. (2005). Self-assembled
monolayers of thiolates on metals as a form of nanotechnology. Chem. Rev..

[54] Zhang S., Dedovets D., Feng A., Wang K., Pera-Titus M. (2022). Pickering
interfacial catalysis for aerobic alcohol oxidation in oil foams. J. Am. Chem. Soc..

[55] Zhang S., Dedovets D., Pera-Titus M. (2022). Oil foams
stabilized by POSS/organosilica
particle assemblies: application for aerobic oxidation of aromatic
alcohols. Journal of Materials Chemistry A.

[56] Feng A., Dedovets D., Zhang S., Sha J., Schwiedernoch R., Gao J., Gu Y., Pera-Titus M. (2022). Foams Stabilized by AquivionTM PFSA:
Application to Interfacial Catalysis for Cascade Reactions. Advanced Materials Interfaces.

[57] Feng A., Dedovets D., Gu Y., Zhang S., Sha J., Han X., Pera-Titus M. (2022). Organic foams
stabilized by Biphenyl-bridged organosilica
particles. J. Colloid Interface Sci..

