# Supplementary material for: Surface-Active Catalysts for Interfacial Gas–Liquid–Solid Reactions
Source: Acc Mater Res. 2025 May 9;6(6):720–9. doi: 10.1021/accountsmr.5c00026 (PMC12216235; doi:10.1021/accountsmr.5c00026)
Supplement: Supplementary file 1 [file mr5c00026_si_001.pdf]

Supporting materials

Surface-Active Catalysts for Interfacial Gas-Liquid-Solid Reactions

Kang Wang, Badri Vishal and Marc Pera-Titus*

Cardiff Catalysis Institute, School of Chemistry, Cardiff University, Main Building, Park Place, Cardiff CF10 3AT, UK

Corresponding Author. E-mail: peratitusm@cardiff.ac.uk

Table of Contents

Figure S1. Energy plots: **(a)** evolution of adsorption energy of a single particle against the contact angle, **(b)** free energy of foam formation against the contact angle for solvents with γ_{GL} = 74 mN·m^{-1} and γ_{GL} = 35 mN·m^{-1}. Plots in **(a)** and **(b)** performed for a particle diameter of d_p = 300 nm and 5-mL gas volume.

Table S1. Advantages and drawbacks of synthetic methods of surface-active particles.

Thermodynamics of Particle Self-Assembly at the G-L Interface and Foam Formation

Bubbles are intrinsically unstable when dispersed in a liquid because the generation of interfacial surface area increases the free energy of the G-L system. The energy required to generate bubbles is a function of the G-L interfacial tension and is promoted for polar liquids. This energy can be counterbalanced by the interfacial adsorption of particles, decreasing the total free energy of the system towards less positive values. This requires first the dispersion of particles in the liquid medium, which depends on their wetting properties. As a rule, particles are more easily wetted in liquids with lower surface tension (<30 mN/m), making it difficult to migrate to the interface, even when the particles possess low surface energy.

The free energy of adsorption of a single, spherical particle at the G-L interface, attributable to changes in interface areas and contact lines, $\Delta_{int}G_p$, can be expressed by Eq 1 earlier developed by Aveyard and Clint by comparison of the free energy of a particle adsorbed at the G-L and the free energy of a particle dispersed in the liquid phase[1, 2]

$$\Delta_{int}G_p = -\frac{\pi}{4}d_p^2\,\gamma_{GL}\left(d_p/D_G\right)\left[(1 \pm cos\,\theta)^2 - \frac{4\tau_{GL}}{d_p\gamma_{GL}\sin\theta}(1 \pm cos\,\theta)\right] \quad (1)$$

where θ is the 3-phase interfacial contact angle (measured through the liquid phase), d_p is the particle diameter, γ_{GL} is the effective G-L interfacial tension that depends on the curvature of the G-L interface (i.e. d_p/D_G ratio, with D_G = swollen bubble diameter),[3] τ_{GL} is the line tension. The (+) and (-) sign in brackets refers to particle removal into the bulk gas and liquid phase, respectively. Eq 1 can be corrected with terms including electrostatic and van der Waals interactions between the adsorbing particles and the particle film. These interactions are often repulsive and can contribute by 20-300kT to $\Delta_{int}G_p$ at high particle coverages (0.97-0.99) for contact angles in the range 50-150°.[4]

If d_p/D_G <<0.1 and $\tau << d_p\,\gamma_{GL}$, Eq 1 can be simplified to the well-known expression that is commonly reported in reviews and manuals:

$$\Delta_{int}G_p = -\frac{\pi}{4}d_p^2\,\gamma_{GL}(1 \pm cos\,\theta\,)^2 \quad (2)$$

As inferred from Eq 1-2, the optimal contact angle for single particle adsorption is 90°. Positive line tensions reduce the length of the contact line and push the contact angle far from 90°, whereas negative line tensions shift the contact angle towards 90°. The line tension exerts an important effect on $\Delta_{int}G_p$ (**Figure S1a**). Keeping the particle diameter and surface tension at constant value (d_p = 300 nm, γ_{GL} = 74 or 35 mN.m^{-1}), the window of contact angles expands for negative line tensions that stabilize bubbles. In contrast, for positive line tensions, the window of contact angles retracts, and the bubbles are destabilized. The effect of the line tension is magnified at lower particle diameter.

The free energy of foam formation can be computed as the product of the free energy of formation of a particle-coated bubble, ΔG_{bubble}, and the number of bubbles, $n_{bubbles}$, by using the expression earlier proposed by Kralchevsky and co-workers by assuming that bubbles behave as hard spheres[5]

$$\Delta G_{foam} = n_{bubbles}\,\Delta G_{bubble} \quad (3)$$

with $n_{bubbles} = \frac{3V_G}{\pi D_G^2}[1 + \varepsilon\varphi_a f(\theta)]$ and $\Delta G_{bubble} = 4\frac{\varphi_a}{\varepsilon^2}[1 + 2\varepsilon\,cos\theta]\left(\pi D_G^2\gamma_{GL} + \Delta_{int}G_p\right)$ (4,5)

In Eqs 3-5, V_G is the gas volume, $f(\theta)$ = [1-cos(θ)]^2 [2+cos(θ)], φ_a ranges within 0 and 0.907 (the latter value referring to a closely packed monolayer), and $\varepsilon = r/R_G$. As mentioned in section 4.1, parameter φ_a plays an important role in Catalysis, since it defines available zones between adsorbed particles with potentially enhanced G-L miscibility.

The plot of ΔG_{foam} against the contact angle shows a minimum at 90° and increases sharply either by decreasing θ to 0°, or by increasing θ to 180°. At constant particle diameter, ΔG_{foam} exhibits positive values except for a narrow window of contact angles around 90° that increases at lower interfacial tensions and for negative line tensions larger than -5000 pN (see example in **Figure S1b** for d_p = 300 nm). This observation points out that, at typical conditions of catalytic reactions, interfacial self-assemblies of particles result in *metastable* foams and that an energy input is necessary to boost foaming before or during the reaction. The foam stability can be promoted using small particles (<100 nm), magnifying line tension effects.[6] The genesis of *metastable* foams is an asset for G-L-S reactions, since this can allow gas regeneration near the catalytic active sites by particle disassembly-reassembly at the G-L interface during the reaction.

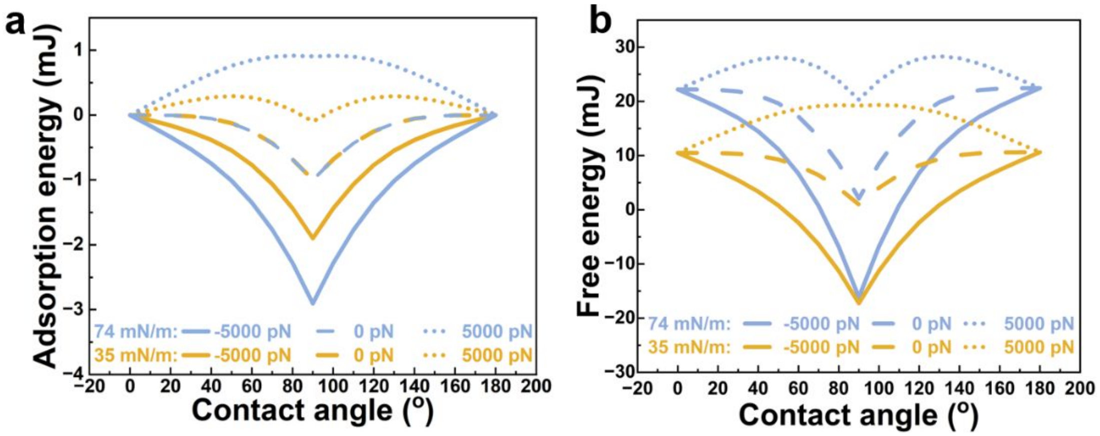

Figure S1. Energy plots: **(a)** evolution of adsorption energy of a single particle against the contact angle, **(b)** free energy of foam formation against the contact angle for solvents with γ_{GL} = 74 mN·m^{-1} and γ_{GL} = 35 mN·m^{-1}. Plots in **(a)** and **(b)** performed for a particle diameter of d_p = 300 nm and 5-mL gas volume.

Dynamics of Particle Self-Assembly at the G-L Interface and Foam Formation

The dynamics of particle adsorption at the G-L interface is influenced by an adsorption barrier (F) that depends primarily on the charge of particles already adsorbed. The extent of the adsorption barrier is associated with the disjoining pressure of the liquid film, represented as Π(h), which varies with the distance, h, between the particle and the interface according to the expression

$$F \approx 2\pi r \int_h^\infty \Pi(h) dh \qquad (6)$$

The existence of an energy barrier for particle adsorption relies on the fact that charged particles approaching a neutral interface between two media with different dielectric constants experiences repulsive image-charge forces.[7] Electrostatic interactions affect the deformation at one or two Debye lengths from the particle. The particle size increases the time needed to stabilize bubbles. The adsorption time also depends on the foaming method that impacts on the hydrodynamics of the G-L system.

The diffusion coefficient for a spherical particle subjected to Brownian motion, *D*, can be expressed by the Stokes-Einstein equation[8]

$$D = \frac{k_B T}{3\pi \eta_L d_p} \qquad (7)$$

where k_B is the Boltzmann constant, T is the temperature, and η_L is the liquid viscosity.

From Eq 7, a first estimate of the time required for the particles to diffuse a distance, h, from one bulk phase to the interface can be obtained using the following expression

$$t \approx \frac{h^2}{D} \quad (8)$$

According to Fick's law, the particle adsorption time decreases at higher particle concentration.[61] The adsorption time decreases for particles with isotropic shapes, but anisotropic shapes and non-uniform surface composition can discourage particle diffusion.[9] Particle diffusion can be enhanced by pre-adsorbing a surfactant/polymer. An additional energy source, such as ultrasound, can further promote particle adsorption.

Because of the high-energy desorption barriers and interparticle interactions, bubble coalescence can be limited over time. Indeed, if the energy required for foam formation is high, the surface coverage is only partial ($\varphi_a < 1$). As a result, bubbles can coalesce only until a compact monolayer ($\varphi_a = 1$) with particle reorientation and rotation,[10-12] which slows adsorption kinetics.[13, 14] By adjusting the particle interactions, it is possible to speed up the adsorption kinetics at high φ_a.[15] As a rule, particle reorientation is discouraged when for non-spherical particles. Besides, restriction of internal dynamics ('jamming') due to surface roughness can occur during particle adsorption, especially for smaller bubbles.

The thickness of particle-stabilized interfaces is much larger than that of interfaces stabilized by surfactants, being at least equal to the particle size for $\varphi_a = 1$. As a result, particle-stabilized foams can exhibit long-lasting large interfacial contact areas and short diffusion paths, allowing fast mass/heat transfer. Positive line tensions may exclude particles from the G-L interface.[1, 4] These properties are of significance for engineering the interfacial microenvironment in catalytic reactions.

Table S1. Advantages and drawbacks of synthetic methods of surface-active particles.

Synthesis method	Advantages	Drawbacks
Post-grafting	**Versatility:**<br>Enables functionalization of pre-existing particles with specific surface-active groups allowing tailored surface properties for a wide range of applications.	**Compatibility issues:**<br>Requires pre-formed particles with specific functional groups, which may not always be readily available.
Co-precipitation	**Simplicity:**<br>A straightforward method that combines reactants to simultaneously form and functionalize particles, simplifying the synthesis process.<br>**Scalability:**<br>Well-suited for large-scale production due to its simplicity and cost-effectiveness.	**Poor control over particle properties:**<br>Size, morphology, and surface composition may be difficult to precisely control.<br>**Reproducibility challenges:**<br>Small variations in synthesis conditions can lead to inconsistencies between batches.
Bottom-up synthesis	**High customizability:**<br>Enables precise engineering of particle size, shape, and surface characteristics during synthesis.<br>**Tailored properties:**<br>Allows the integration of complex functionalities, such as stimuli-responsive groups or hierarchical structures.	**Complexity:**<br>Often requires advanced techniques, specialized equipment, and careful optimization of reaction parameters.<br>**Limited scalability:**<br>The detailed control often achieved at the laboratory scale may not translate easily to industrial-scale production.

References

1. Aveyard, R.; Clint, J. H., Particle wettability and line tension. Journal of the Chemical Society, Faraday Transactions 1996, 92 (1), 85-89.
2. Aveyard, R.; Clint, J. H., Liquid droplets and solid particles at surfactant solution interfaces. Journal of the Chemical Society, Faraday Transactions 1995, 91 (17), 2681-2697.
3. Levine, S.; Bowen, B., Capillary interaction of spherical particles adsorbed on the surface of an oil/water droplet stabilized by the particles. Part I. Colloids and Surfaces 1991, 59, 377-386.
4. Aveyard, R.; Clint, J. H.; Horozov, T. S., Aspects of the stabilisation of emulsions by solid particles: Effects of line tension and monolayer curvature energy. Physical Chemistry Chemical Physics 2003, 5 (11), 2398-2409.
5. Kralchevsky, P.; Ivanov, I.; Ananthapadmanabhan, K.; Lips, A., On the thermodynamics of particle-stabilized emulsions: curvature effects and catastrophic phase inversion. Langmuir 2005, 21 (1), 50-63.
6. Sacanna, S.; Kegel, W.; Philipse, A., Thermodynamically stable pickering emulsions. Physical review letters 2007, 98 (15), 158301.
7. Mbamala, E.; Von Grünberg, H., Effective interaction of a charged colloidal particle with an air-water interface. Journal of Physics: Condensed Matter 2002, 14 (19), 4881.
8. Zoueshtiagh, F.; Baudoin, M.; Guerrin, D., Capillary tube wetting induced by particles: towards armoured bubbles tailoring. Soft matter 2014, 10 (47), 9403-9412.
9. Gao, W.; Jiao, Y.; Dai, L. L., The effects of size, shape, and surface composition on the diffusive behaviors of nanoparticles at/across water-oil interfaces via molecular dynamics simulations. Journal of Nanoparticle Research 2016, 18, 1-11.
10. Rezvantalab, H.; Drazer, G.; Shojaei-Zadeh, S., Molecular simulation of translational and rotational diffusion of Janus nanoparticles at liquid interfaces. The Journal of chemical physics 2015, 142 (1).
11. Luu, X.-C.; Yu, J.; Striolo, A., Ellipsoidal Janus nanoparticles adsorbed at the water-oil interface: some evidence of emergent behavior. The Journal of Physical Chemistry B 2013, 117 (44), 13922-13929.
12. Gao, H.-M.; Lu, Z.-Y.; Liu, H.; Sun, Z.-Y.; An, L.-J., Orientation and surface activity of Janus particles at fluid-fluid interfaces. The Journal of chemical physics 2014, 141 (13).
13. Schwenke, K.; Isa, L.; Del Gado, E., Assembly of nanoparticles at liquid interfaces: crowding and ordering. Langmuir 2014, 30 (11), 3069-3074.
14. Luu, X.-C.; Yu, J.; Striolo, A., Nanoparticles adsorbed at the water/oil interface: coverage and composition effects on structure and diffusion. Langmuir 2013, 29 (24), 7221-7228.
15. Schwenke, K.; Del Gado, E., Soft repulsive interactions, particle rearrangements and size selection in the self-assembly of nanoparticles at liquid interfaces. Faraday discussions 2015, 181, 261-280.
